# Initial PCV Chemotherapy Followed by Radiotherapy Is Associated With a Prolonged Response But Late Neurotoxicity in 20 Diffuse Low-Grade Glioma Patients

**DOI:** 10.3389/fonc.2022.827897

**Published:** 2022-03-04

**Authors:** Marie Blonski, Tiphaine Obara, Cyril Brzenczek, Celso Pouget, Céline Dillier, Mylène Meyer, Laura Lavigne, Natacha Forthoffer, Aurélie Broussois, Guillaume Gauchotte, Marie-Hélène Baron, Fabien Rech, Sophie Mézières, Yann Gaudeau, Antoine Verger, Guillaume Vogin, René Anxionnat, Jean-Marie Moureaux, Luc Taillandier

**Affiliations:** ^1^ Department of Neurology, Neurooncology Unit, CHRU, Nancy, France; ^2^ Centre de Recherche en Automatique Nancy France - UMR 7039 - BioSiS Department, Faculty of Medicine, Université de Lorraine, Vandoeuvre-lès Nancy, France; ^3^ Department of Pathology, CHRU, Nancy, France; ^4^ Centre de ressources Biologiques, BB-0033-00035, CHRU, Nancy, France; ^5^ Department of Neurosurgery, CHRU, Nancy, France; ^6^ Department of Mathematics, Elie Cartan Institute, Nancy, France; ^7^ INRIA Biology, Genetics and Statistics, Nancy, France; ^8^ Department of Nuclear Medicine and Nancyclotep Imaging Platform, CHRU, Nancy, France; ^9^ IADI, INSERM U1254, Université de Lorraine, Vandoeuvre-lès Nancy, France; ^10^ Department of Radiation Therapy, Baclesse Radiation Therapy Center, Esch/Alzette, Luxembourg; ^11^ UMR 7635 CNRS, IMoPA Biopole Lorraine University Faculty of Medicine, Université de Lorraine, Vandoeuvre-lès-Nancy, France; ^12^ Department of Neuroradiology, CHRU, Nancy, France

**Keywords:** diffuse low-grade glioma, quality of life, neurocognition, neurotoxicity, kinetics, surgery, radiotherapy, chemotherapy

## Abstract

**Background:**

Study RTOG 9802 in high-risk diffuse low-grade gliomas (DLGGs) showed the potential synergistic effect on survival of the procarbazine, CCNU, and vincristine (PCV) radiotherapy (RT) combination. Limited data on long-term neurocognitive impact and quality of life (QoL) have yet been reported.

**Patients and Methods:**

We described a monocentric series of patients treated at first line by the combination of PCV immediately followed by RT between January 01, 1982 and January 01, 2017. Radiological data were collected and included volume, velocity of diametric expansion (VDE), and MRI aspects. Long-term neurocognitive and QoL were analyzed.

**Results:**

Twenty patients fulfilled the eligibility criteria. The median response rate was 65.1% (range, 9.6%–99%) at the time of maximal VDE decrease corresponding to a median volume reduction of 79.7 cm^3^ (range, 3.1 to 174.2 cm^3^), which occurred after a median period of 7.2 years (range, 0.3–21.9) after the end of RT. An ongoing negative VDE was measured in 13/16 patients after the end of RT, with a median duration of 6.7 years (range, 9 months–21.9 years). The median follow-up since radiological diagnosis was 17.5 years (range, 4.8 to 29.5). Estimated median survival was 17.4 years (95% CI: 12; NR). After a long-term follow-up, substantial neurotoxicity was noticed with dementia in six progression-free patients (30%), leading to ventriculo-peritoneal shunt procedures in three, and premature death in five. Thirteen patients (65%) were unable to work with disability status. Successive longitudinal neurocognitive assessments for living patients showed verbal episodic memory deterioration.

**Conclusions:**

PCV-RT combination seems to have not only an oncological synergy but also a long-term neurotoxic synergy to consider before initial therapeutic decision.

## Introduction

Diffuse low-grade gliomas (DLGGs) are slow-growing brain tumors, which inevitably evolve to high-grade glioma leading to death. DLGGs affect socially active patients (median age at diagnosis of 40 years). The aim of the oncological management is to delay as much as possible the anaplastic transformation while preserving quality of life and neurocognition. The optimal treatment sequence remains controversial. Whereas functional surgery has to be prioritized given its impact on function and survival (1), the place of chemotherapy remains debated. A recent study underlines its benefit on survival (2). Especially, its timing and articulation with radiotherapy remain discussed. Radiotherapy alone presents the same results in terms of survival regardless of treatment timing (early or late) (3). However, radiotherapy and chemotherapy together seem to have a synergistic oncological effect as reported in Radiation Therapy Oncology Group (RTOG) 9802 (2) and RTOG 0424 (4). Yet, no robust neurocognitive (MMSE) (5) and quality of life (QoL) (European Organization for the Research and Treatment of Cancer Quality of Life Questionnaire (EORTC QLQ-C30) + BN20 questionnaire) data are available for this population, where many patients may live more than 10 years, thus being at risk of delayed neurocognitive toxicity. Despite the lack of these data and this potential risk, a trend towards the generalization of this strategy is noted (6).

In this context, we analyzed within our local database (7), the subgroup of patients treated by procarbazine, CCNU, and vincristine (PCV) immediately followed by radiotherapy whatever surgical status. We report here the long-term follow-up of 20 patients, with clinical, radiological, kinetics, neurocognitive and QoL data.

## Patients and Methods

### Selection Criteria

**Figure 1:**
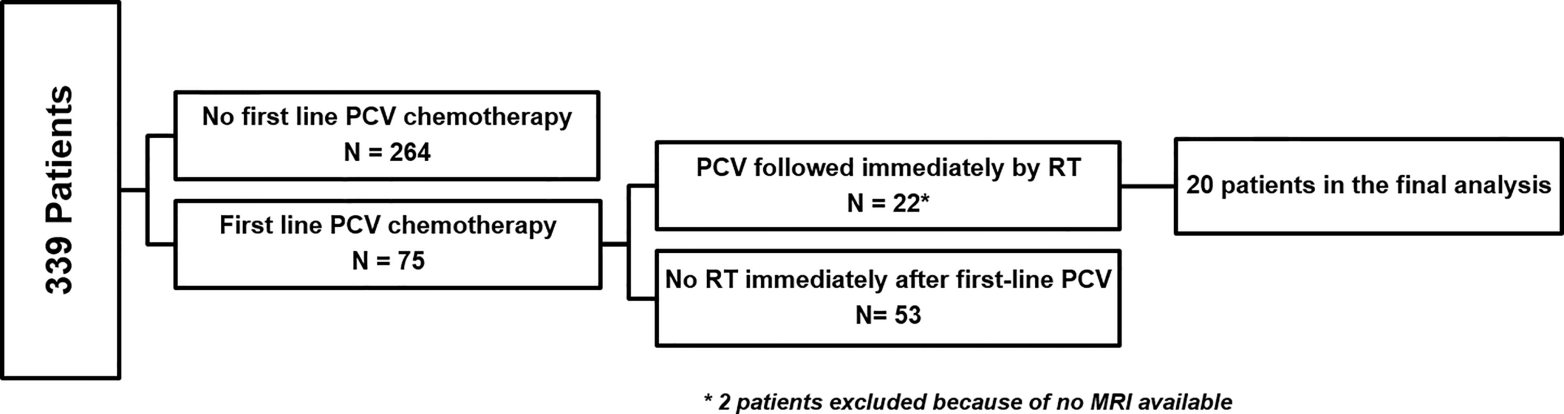
Flowchart of patient selection from the database.

We requested on our database including 339 DLGG patients diagnosed between January 01, 1982 and January 01, 2017 (7) according to the following criteria: patients older than 18 years at diagnosis; histological DLGG diagnosis before PCV chemotherapy; patients treated by the sequence PCV followed by radiotherapy at first-line adjuvant treatment without anaplastic transformation; validation of the therapeutic strategy in a multidisciplinary meeting; and delay between the end of PCV and radiotherapy onset of less than 6 months (**Figure 1**).

### Clinical and Treatment Data

Clinical data included age, sex, vascular risk factors, symptoms at diagnosis, longitudinal KPS score, seizure and dedicated treatments during the follow-up, dates of biopsy or surgical removal, and quality of resection.

The protocol consisted in the standard PCV schedule (lomustine 110 mg/m^2^ on day 1, procarbazine 60 mg/m^2^ on days 8–21, and vincristine 1.4 mg/m^2^, maximum 2 mg on days 8 and 29, in 6-week cycles, for a maximum of 6 cycles). Toxicity and adverse effects were graded according to the NCI-CTC v4.0. Concerning radiotherapy, timing, total dose, and dose per fraction were collected.

### Neuroimaging Data

Tumor volumes were measured with the opensource medical imaging software Osirix Lite when MRI available at diagnosis and during the follow-up period (before, during and after the sequence PCV followed by radiotherapy).

When only printed images were available, tumor volumes were estimated with the three-diameter technique by the ellipsoid approximation (8). The measurements were performed on T2-weighted or fluid attenuation inversion recovery (FLAIR) sequences by one investigator (MB).

The response rate was calculated using the following formula: [Vpre − Vpost]/Vpre, where Vpre and Vpost correspond to the “pre- and postchemotherapy/radiotherapy/combined treatment” volumes, respectively.

The quantitative analysis of the imaging tumor growth (i.e., the velocity of diametric expansion (VDE) was measured on serial MR examinations for each patient over the follow-up period. The mean tumor diameter (MTD) was deduced from the tumor volume (V) through the formula MTD = (2 × V)^1/3^. The VDE (i.e., the glioma growth curve) was plotted as a function of MTD over time using a method previously recommended (8). VDE was measured before, during, and after PCV chemotherapy and radiotherapy. VDE after chemotherapy corresponds to the VDE before radiotherapy. The response to PCV chemotherapy, radiotherapy, and combined treatment was respectively assessed by the quantitative radiological changes of tumor volume and VDE, before and after each sequence.

Topography and contrast enhancement at radiological diagnosis were noticed as absent, faint and patchy, nodular like, and ring like as previously described (9). Malignant transformation was proved histologically or suspected *via* the occurrence of a nodular-like or ring-like contrast enhancement (9).

To assess MRI abnormalities associated with delayed toxicity, different criteria were analyzed: (a) white matter FLAIR or T2-weighted lesions according to Fazekas scale (10), (b) cortico-subcortical brain atrophy assessed and graded on a 0–3-point scale, no atrophy (grade 0), some atrophy and widening of sulci (grade 1), pronounced atrophy and loss of volume in the gyri (grade 2), and end-stage atrophy with knife blade shapes (grade 3); and (c) cerebral small vessels disease signs, including lacunae of presumed vascular origin, perivascular spaces and cerebral microbleeds (11).

### Pathological and Molecular Data

All tumors were centrally reviewed by experiment neuropathologists (GG, CP) and were classified according to the 2016 WHO classification (12). Paraffin sections of 5 μm thickness were immersed in a 10-mM sodium citrate buffer (pH 6) for 20 min at 97°C for dewaxing and antigen retrieval. The following primary antibodies were used: Ki-67 (1/200; mouse monoclonal, MIB-1, Dako Cytomation, Glostrup, Denmark), Alpha-internexin (mouse monoclonal, 2E3, Invitrogen, Carlsbad, CA, USA), P53 (mouse monoclonal, DO-7, Dako Cytomation, Glostrup, Denmark), IDH1 R132H (1/70, mouse monoclonal, H09, Dianova, Hamburg, Germany), ATRX (1/200, rabbit polyclonal, Sigma, St. Louis, MO, USA). Immunohistochemistry was performed with Dako Autostainer Plus (Dako) and the Flex+ Envision revelation system (Dako). The percentage of MIB-1-positive cells was determined by counting cells in continuous microscopic fields at a magnification of ×400. A cutoff at 5% was established between a low and high proliferation index. Samples were considered positive for p53 expression when more than 10% of the nucleus was labeled. Loss of ATRX expression is mutually exclusive with 1p19q codeletion.

### Statistical Analyses

All data analyses were performed using R version 4.0. For descriptive statistics, we used numbers and percentages; for qualitative ones, variables; and for quantitative ones, medians and ranges. Estimated survival and time to TA were calculated from the time of radiological diagnosis until death and anaplastic transformation, respectively. Survival curves were estimated using the Kaplan–Meier method with a 95% confidence interval and differences tested by the log-rank test. Spearman correlation coefficient was used to assess any possible association between numerical variables, and a one-way ANOVA was performed to determine the relationship between a numerical variable and a categorical one. To assess prognostic factors, univariate analysis was first performed using log-rank tests for qualitative variables and univariate Cox models for quantitative ones. Factors with p value ≤ 0.05 were considered statistically significant.

### Neurocognitive and Quality- of- Life Assessments

A set of neuropsychological examinations was carried out in progression-free patients. The set used in the present study was the same as that detailed in a previous report (13). It was aimed to evaluate global efficiency, laterality, and seven cognitive domains: information processing/psychomotor speed, attention, episodic memory (verbal and nonverbal), verbal working memory, language, visuospatial abilities, and executive functions.

A complete set took from 90 to 120 min. Scores on all test parameters were converted to z-scores by comparison with the mean and standard deviation of a reference based on healthy control groups who were matched individually for age, sex, and educational level. All tests have been standardized for the French population. A deficient score was defined as a value of at least two standard deviations (SDs) under the corresponding value for a healthy control group or inferior to the fifth percentile.

At the end of neuropsychological evaluation, several QoL questionnaires were delivered to the patients: the EORTC QLQ-C30 questionnaire associated to the Brain Module BN-20 for quality of life (14), the Beck Depression Inventory (BDI) (15) for symptoms of depression, the State-Trait Anxiety Inventory (STAI-A, STAI-B) (16) for symptoms of anxiety, the Multi-dimensional Fatigue Inventory (MFI) (17) for fatigue assessment, and a socioprofessional questionnaire dedicated to this study. For purposes of analysis, all data were linearly transformed into a 0–100 range.

## Results

### Clinical, Histological, and Molecular Data

Twenty patients fulfilled the eligibility criteria. Their main characteristics are listed in **Table 1**.

**Table 1:** Characteristics of the study population (*N* = 20).

Clinical features at radiological diagnosis	
**Median age (year)**	40 (24–62)
Sex (M/F)	11/9
Median KPS (%)	90 (70–100)
Vascular risk	3
**Symptoms at diagnosis**	
Partial seizure	12
Secondarily generalized seizure	6
Intracranial hypertension	2
**Tumor location (right/left)**	
Frontal	11 (3/8)
Temporal	5 (1/4)
Parietal	2 (0/2)
Fronto-temporal	1 (1/0)
Temporo-parietal	1 (0/1)
**Gadolinium enhancement at diagnosis**	
None	10
Patchy and faint	7
Nodular	3
**Previous surgery (or biopsy)** **^a^**	
Biopsy	14
Surgical removal	6
Partial	1
Subtotal	5
**Histological and molecular subtypes**	
IDH mutated/codeleted tumors	10
IDH mutated/noncodeleted tumors	9
IDH wild-type tumor	1
Median Ki-67 proliferation index (%)	3 (0.5–10)
**At combined treatment onset**	
Median age at PCV onset	43 (range, 29–62)
Median delay between radiological diagnosis and PCV onset	11months (range, 0.4–82)
Median KPS at PCV onset	90 (range, 70–100)

M, male; F, female; KPS, Karnofsky Performance Status.

a Surgical procedures performed before the combined therapy (PCV chemotherapy following by radiotherapy).

### PCV Chemotherapy Followed by Radiotherapy

PCV chemotherapy was proposed to 14 patients (70%) at diagnosis (nine patients with deemed unresectable tumor and five after surgical removal) and to 6 patients (30%) at progression, with a median time from radiological diagnosis of 11.3 months (range, 0.4–82.2) and a median time from surgical procedure of 9 months (range, 7–8). All patients were considered at high-risk DLGGs given their RTOG 9802 classification (2).

The median number of PCV cycles was 4 (range, 2–6). Four patients (20%) received less than 4 cycles and eight patients (40%) received six cycles. The regimen was associated with grades 3–4 hematological toxicity in nine patients (45%), with a discontinuation of the treatment in four patients. One patient exhibited significant neuropathy. No correlation was highlighted between PCV toxicity and age or antiepileptic drugs (enzymatic inhibitor or inductor).

Median KPS before PCV and at the end of PCV onset were respectively 90% (range, 60%–100%) and 80% (range, 70%–90%). Radiotherapy was initiated after a median of 2.4 months (range, 0.4–5.1) after PCV discontinuation. Median total dose of radiotherapy was 54 Gy (range, 45–60). Ten patients (50%) received a fraction per dose of 1.8 Gy and ten patients (50%) a fraction per dose of 2 Gy. Median KPS at the end of the combined treatment was 80% (range, 70%–90%).

### Tumor Kinetics and Volumes

**Figure 2:**
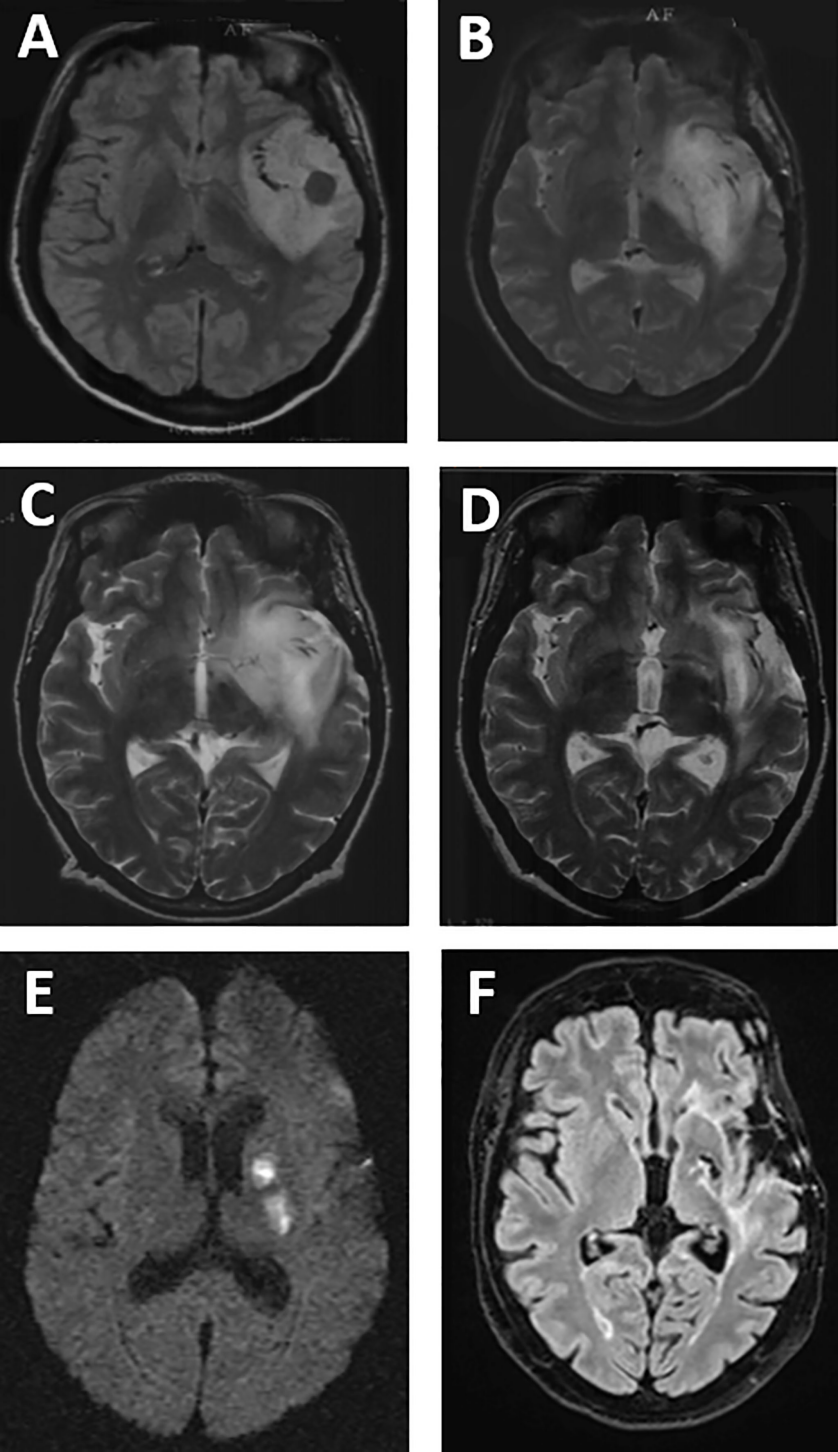
Example of MRI response and complication, in left temporal IDH/noncodeleted DLGG, in FLAIR **(A, F)**, T2-weighted **(B–D)**, and diffusion-weighted imaging **(E)**. Precise **(A)** at diagnosis and after biopsy. **(B)** MRI before PCV and 3 months after partial resection. **(C)** MRI after PCV (before RT). **(D)** MRI after RT (after PCV + RT). **(E)** MRI 6 years after the end of PCV + RT (stroke lesions). **(F)** MRI 19 years after the end of PCV + RT and 25 years after diagnosis.

The evolution of tumor volumes over time is detailed in **Table 2**. PCV induced tumor shrinkage in 17/18 cases with a median response rate of 20% (range, 0%–81%) corresponding to a median volume reduction of 30 cm^3^ (range, 0–170). The only patient who presented tumor progression under PCV had a limited volume before PCV (10 cm^3^). This patient and two others presented a tumor growth (positive VDE) between the end of PCV and RT onset, but tumor volume finally decreased for the three during RT (negative VDE) with prolonged and ongoing stabilization. RT allowed tumor volume decrease in 18/18 available (100%) with a median response rate of 13% (range, 0%–65%). At the end of the combined treatment, 17/18 patients (95%) experienced a volume diminution with a median of 41 cm^3^ (range, 0–168) corresponding to a median response rate of 31% (range, 0%–80%). An example of radiological response is shown in **Figure 2**. Median response rate was 29% (range, 0% to 81%) in IDH-mutated/codeleted tumors, 15% (range, 1% to 50%) in IDH-mutated/non-codeleted tumors and 5% in the only one IDHwt tumor.

**Table 2:** Tumor volumes before, during, and after the combined therapy.

	Volume at diagnosis (cm^3^)	Volume before PCV (cm^3^)	Volume after PCV (before RT) (cm^3^)	Volume variation induced by PCV % (cm^3^)	Interval between end of PCV and RT (month)	Volume before RT (cm^3^)	Volume after RT (cm^3^)	Volume variation induced by RT % (cm^3^)	Volume variation induced by PCV + RT % (cm^3^)
	*N* = 18	*N* = 18	*N* = 18	*N* = 18		*N* = 18	*N* = 19	*N* = 18	*N* = 18
Mean ± SD	95 ± 42	112 ± 57	80 ± 58	24 ± 17^a^	2.7 ± 0.8	82 ± 42	76 ± 47	17 ± 13	36 ± 17
				(44 ± 28)				(14 ± 13)	(47 ± 31)
Median	104	119	84	20^a^ (30)	2.4	85	75	13 (7)	31 (41)
Range	13–190	1–243	1–182	0–81^a^ (0–170)	0.4–5.1	1–182	1–175	0–65 (0–168)	0–80 (0–168)

VDE, volumetric diametric expansion; RT, radiotherapy; SD, standard deviation.

a The only patient who presented tumor progression under PCV was not reported.

**Figure 3:**
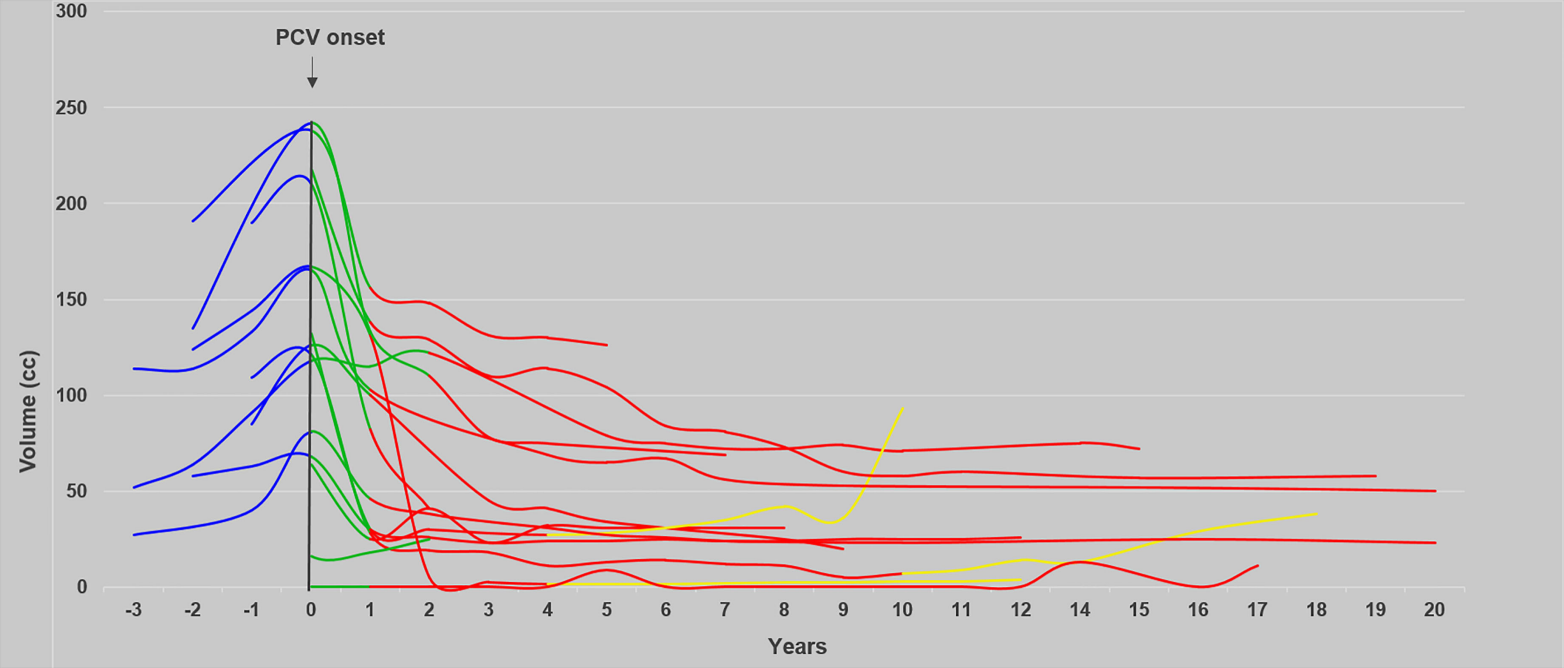
Evolution of the tumor volume over time before, during, and after the sequence PCV followed by RT. The evolution of tumor volume before (*n* = 10), during (*n* = 16), and after (*n* = 16) the sequence PCV followed by RT and at progression is shown in blue, green, red, and yellow, respectively.

The detailed evolution of kinetics is available in **Table 3**. The evolution of tumor volume before, during, and after the combined treatment is represented in **Figure 3**.

**Table 3:** Tumor kinetics before, during, and after the combined therapy.

	Spontaneous VDE (before PCV) (mm/year)	VDE during PCV (mm/year)	VDE after PCV (before RT) (mm/year)	VDE during RT (mm/year) *N* = 18	VDE after RT (mm/year) *N* = 16	Total duration of negative VDE (years) *N* = 14	Duration of negative VDE after end of RT (years) *N* = 14
	*N* = 11	*N* = 18	*N* = 18				
Mean ± SD	4.9 ± 2.1	−5 ± 4.5	−0.4 ± 7	−6.2 ± 4.9	−2.1 ± 2.6	7.6 ± 4.4	7.4 ± 4.3
Median	3.8	−3.4	−3	−4.5	−1.7	6.4	5.6
Range	2.3 to 11.3	−29.4 to 6.1	−8.7 to 36	−26.1 to 0	−9.9 to 5.8	0.5–22.2	0.8–21.9

VDE, volumetric diametric expansion; SD, standard deviation.

Before PCV, two patients (10%) presented a VDE of 8 mm/year or more (high-rate group) (18). During PCV, the VDE decreased in 17/18 patients with a median of −3.5 mm/year (range, −0.4 to −29.4). At the end of PCV and before RT onset, VDE decrease was observed in 15/18 patients with a median VDE of −3.5 mm/year (range, −0.4 to −8.7). During RT, VDE decrease was reported in 18/18 patients (100%) with a median of −4.5 mm/year (range, 0 to −26.1). After the combined treatment, a persistent VDE decrease was observed in 13/16 patients (80%) with a median decrease of −2.3 mm/year (range, −0.4 to −9.9). After visual inspection, VDE was probably overestimated for these three VDE-positive patients after the combined treatment because of radiation-induced MRI changes. These three patients had no recurrence during the follow-up period. Moreover, one patient treated with three cycles of PCV and a total dose of 56 Gy experienced a transient positive VDE 14 months after the end of RT due to radionecrosis. This progression-free patient then exhibited a persistent negative VDE but developed severe cognitive disturbances leading to premature death.

The median duration of the negative VDE was 8.2 years (range, 2.3–22.5) after PCV onset, 6.3 years (range, 0.4–22.1) after RT onset, and 6.7 years (range, 0.9–21.9) after the end of the combined treatment.

Median duration of VDE negative after the end of the combined treatment was 8.1 years (range, 0.8 to 8.6) in IDH-mutated/codeleted tumors, 5.6 years (range, 2.5 to 21.9) in IDH-mutated/noncodeleted tumors, and 3.7 years in the IDHwt tumor.

For patients who received 6 PCV cycles (*n* = 8), 4 PCV cycles (*n* = 5), 3 PCV cycles (*n* = 2), and 2 PCV cycles (*n* = 5), the median duration of the negative VDE after PCV onset was respectively 6.9, 10.4, 9.2, and 8.7 years.

The median response rate was 65.1% (range, 9.6%–99%) at the time of maximal MTD decrease corresponding to a median volume reduction of 79.7 cm^3^ (range, 3.1–174.2), which occurred after a median period of 7.2 years (range, 0.3–21.9) after the end of the combined treatment and after 8.1 years (range, 0.33–22.5) after PCV onset. A total of 60% patients demonstrated prolonged response (>3.6 years) after the end of RT without any other oncological treatment.

One patient was operated on 4 months after the end of the combined treatment with a subtotal resection. The tumor residue volume continued to decrease (meaning a negative VDE) 4.3 years after surgical removal.

### Neurotoxicity on MRI

Leuco-encephalopathy appeared in 15 patients (75%) with a median delay of 6.9 years (range, 0.5–11.5) after the end of RT. Leuco-encephalopathy was graded as Fazekas grade 3 in 10 patients, grade 2 in two patients, and grade 1 in three patients. Four (20%) patients presented microbleeds or radiation-induced cavernoma. Six patients (30%) showed enlargement of perivascular spaces. Lacunae of presumed vascular origin were seen in six patients (30%). Among them, two patients were symptomatic and one kept neurologic sequelae with right hemiparesis (**Figure 2**). Cortico-subcortical atrophy was noted in 12 (60%) patients (six patients with grade 1, three with grade 2, and three with grade 3). One patient exhibited radionecrosis 14 months after the end of combined treatment. Five patients (25%) presented hydrocephalus requiring ventriculo-peritoneal shunt in three of them. Median delay between the end of RT and shunt procedure was 6.9 years (range, 3.9–7.1).

### Survival Analyses

At the time of the study, 8 patients (40%) are still alive with a follow-up from 7 to 29 years. Among these 8 patients, 6 patients were not operated on (only biopsy).

The median follow-up since radiological diagnosis was 17.5 years (range, 4.8–29.5), and the median follow-up since the end of RT was 15.6 years (range, 3.7–24). During this period, eight patients (40%) presented malignant transformation 10.6 years (range, 2.6–20) since radiological diagnosis and 6.7 years (range, 2–19) since the end of RT. Oncological treatments at anaplastic transformation consisted in temozolomide chemotherapy in five patients, PCV rechallenge in one patient, surgical removal in one patient, and palliative care for the last one due to altered general condition.

Estimated median survival was 17.4 years (95% CI: 12; NR). On molecular subgroups, estimated survival was 17 years (95% CI: 13; NR) in IDH/codeleted tumors and was not reached in IDH/noncodeleted tumors (95% CI: 12; NR).

On univariate analysis, KPS ≥90 before PCV and total dose of RT >54 Gy were associated with a better prognosis (hazard ratio = 0.68, *p* < 0.05).

On multivariate analysis, only the total dose of RT was significantly associated with a better survival. Estimated survival in patients who received a total dose ≤54 Gy was 13 years (95% CI: 10–NR); whereas, estimated survival was not reached for patients who received a total dose >54 Gy (*p* < 0.05).

### Neurocognitive and Quality-of-Life Data

**Figure 4:**
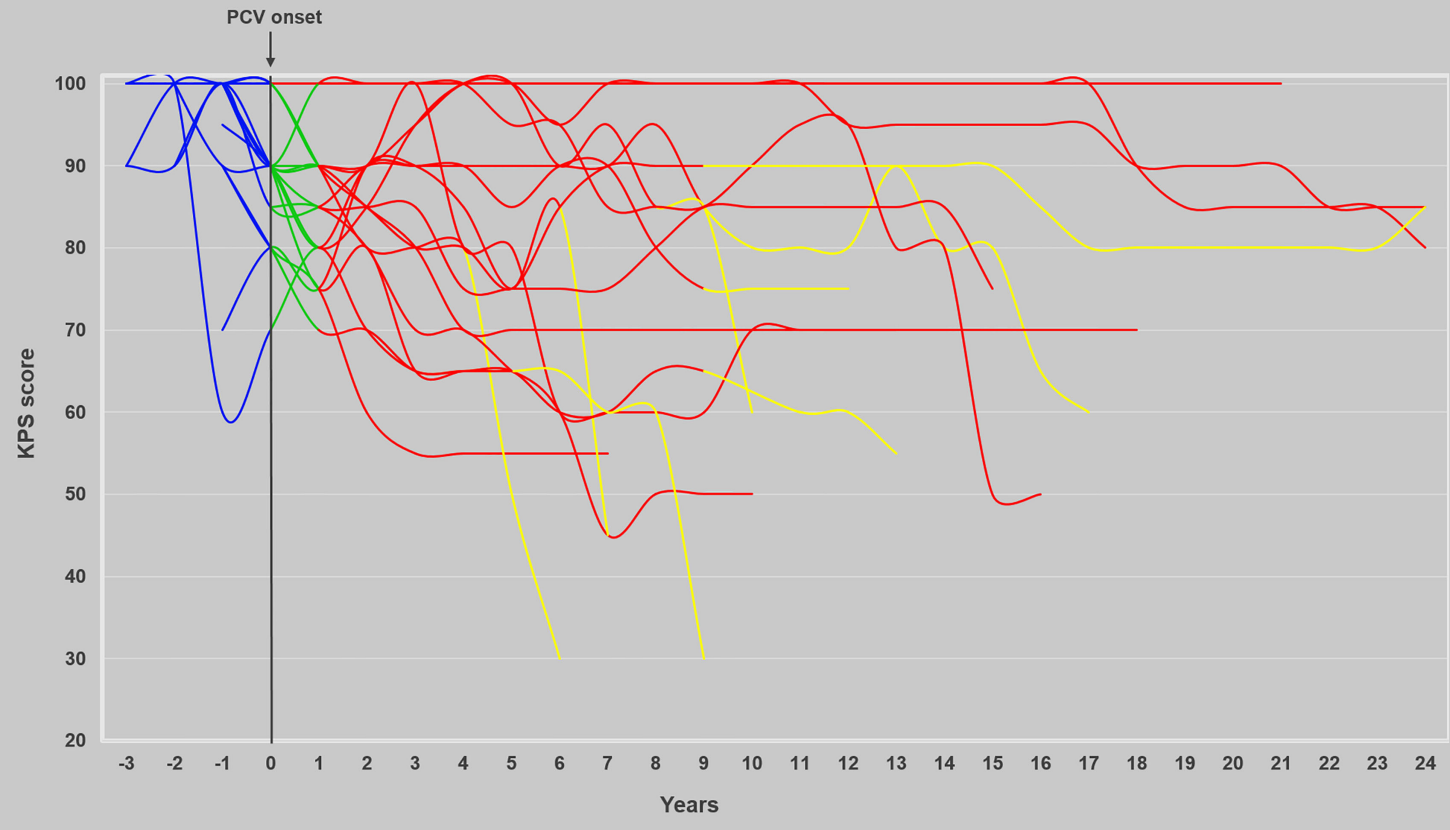
Evolution of KPS over time before, during, and after the sequence PCV followed by RT. The evolution of KPS (*n* = 20) before, during, and after the combined treatment and at progression is represented in blue, green, red, and yellow, respectively.

At the time of the study, eight patients (40%) are still alive with a median KPS of 80% (range, 50%–100%) and a median follow-up since radiological diagnosis of 26.9 years (range, 7.9–29.5). Longitudinal evolution of KPS is represented in **Figure 4**.

Concerning seizure, PCV chemotherapy followed by RT allowed seizure control in all cases and eighteen patients (90%) were seizure free at the end of the combined treatment. Fifteen patients (75%) required only one antiepileptic drug during the follow-up period.

Professionally, two patients continued to work during the combined treatment. After treatment, seven patients (35%) were able to resume professional activity (temporarily for three and long term for four patients, with the same job for five patients, and with an adapted job due to disease or treatment for two patients). After the combined treatment, thirteen patients (65%) were unable to work with disability status linked to disease in five patients, to treatment toxicity in six patients, and to both in two patients.

Concerning cognitive functioning, two patients, still alive, developed severe neurocognitive disorder and became bedridden with dementia for one. One patient remains paretic since an ischemic stroke occurring 7 years after the end of RT (**Figure 2**).

On the global series, twelve patients (60%) died, including six deaths consecutive to tumor progression, five deaths consecutive to treatment neurotoxicity (cognitive deterioration evolving to dementia with no radiological argument in favor of tumor progression, and severe leukoencephalopathy), and one death related to both. We found positive correlation between age at the time of RT and neurotoxicity (dementia, atrophy, hydrocephalus) (*p* < 0.05) but not with sociocultural level, topography, dose per fraction, total dose of RT. Six patients (30%) exhibited severe cognitive impairment. One had vascular risk factors (hypertension, dyslipidemia). One had already mild psychiatric disorders related to alcohol dependence. For these six patients, the median tumor volumes before PCV chemotherapy and before RT were respectively 32 cm^3^ (range, 1–243) and 29 cm^3^ (range, 1–133). The median tumor volumes before PCV chemotherapy and RT in patients without cognitive deterioration were 121 cm^3^ (range, 41–212) and 103 cm^3^ (range, 34–182).

During follow-up, eight patients (40%) were evaluated on neurocognitive functioning with a median age of 55.9 (range, 47.1–64). The median interval after radiological diagnosis was 19.8 years (range, 10.4–23.3) and after RT completion 17.4 years (range, 5.8–17.9). Among the eight patients still alive, six were assessed on neurocognition. One patient refused evaluation, and the last one was unable to be evaluated because of severe neurological condition (bedridden with dementia). In another patient, neurocognitive testing was incomplete due to visual disorder and difficulties in understanding test instructions. Three patients were evaluated on twice with a median interval between the end of RT and the second evaluation of 24.1 years (range, 22.6–24.6), and one patient was evaluated on three times with a delay between the end of RT and the third evaluation of 24.5 years. Six cognitive domains were impaired. The main ones were verbal episodic memory (5 patients), divided attention (5 patients), language abilities (4 patients), working verbal memory (5 patients), and executive functions (5 patients). A second neurocognitive assessment was performed on three patients with a median delay of 6.7 years (range, 4.9–6.8) after the first evaluation, highlighting the development of verbal episodic memory and language impairments in one patient (stable results for the two other patients). A third neurocognitive evaluation was achieved in one nonprogressive patient (at 61.9 years) showing verbal episodic memory and information treatment speed deterioration, 1.9 years after the second evaluation.

Concerning endocrinologic complications, two patients (10%) presented pituitary insufficiency during the follow-up, leading to medically assisted procreation procedures in one patient.

Concerning QOL, questionnaires (EORTC QLQ-C30 + BN20, MFI 20, BDI, and STAY questionnaires) were proposed to the eight survival patients. A first assessment occurred in six patients, with a median interval of 21.9 years (range, 18.9–23.2) after MRI diagnosis and with a median interval of 24.1 years (range, 23.8–24.5) after RT completion. Two patients were unable to understand the grids. A second evaluation occurred in three patients with a median delay of 6.4 years after the first evaluation (range, 6.4–6.8). Three patients refused to complete the second evaluation and two patients were still unable to do so. At the first completion, the median global QOL score was moderately preserved: 70.8% (range, 50%–83.3%). Median role functioning (feeling of independence and socioprofessional life), cognitive, emotional, physical, and social well-being scores were respectively 100% (range, 50%–100%), 74.5% (range, 66.7%–100%), 75% (range, 33.3%–100%), 80.5% (range, 55.5%–93.3%), and 74.5% (range, 50%–100%). Among the general symptoms, the main complains were fatigue (median score, 22.2%; range, 11.1%–55.5%) and sleeping troubles (median score, 33.3%; range, 33.3%–66.7%). Financial impact (median score, 33.3%; range, 0%–66.7%) seemed to have a moderate influence on the QOL. At second QOL evaluation, scores were globally stable apart from median cognitive score (66.7%), which seems to deteriorate.

At first QOL assessment, no patient reached the cutoff of 15 in the inventory for signs or symptoms of depression (BDI); at second assessment, one patient presented significant mood disturbances.

Concerning fatigue, the median total score (global fatigue) was 58% (range, 46%–65%). The median scores for the subscales were as follows: 64% for physical fatigue (range, 49%–69%), 67% for mental fatigue (range, 47%–70%), 73% for reduced activity (range, 47%–80%), and 40% for reduced motivation (range, 20%–60%). Scores between the first and second evaluations were not significantly modified.

Concerning anxiety, no patient presented significant anxiety at the first assessment. Two patients reported moderate level of anxiety at the second.

## Discussion

### Kinetics and Response Duration

To our knowledge, dynamic evaluation of the association of PCV followed by RT has not yet been reported. Several studies have already demonstrated the impact of temozolomide (TMZ) (19), PCV (20), CCNU (21), and RT (22, 23) on DLGG growth kinetics. In the present series, significant and prolonged responses were noticed. During PCV chemotherapy, VDE decrease was observed in 95% of patients with a median of −3.5 mm/year (range, −0.4 to −29.4) with slightly better results observed during a previous study of PCV alone (20) (−10.2 mm/year; range, −1 to −23), TMZ (19) (−9.2 mm/year; range, −0.5 to −39.1), and CCNU (21) (−5.1 mm/year; range, −1 to −8.9). During RT, all patients experienced a VDE decrease.

After the combined treatment, 80% patients had a prolonged negative decrease with a median of −2.3 mm/year (range, −0.4 to −9.9) as previously described after PCV alone (20) (−4 mm/year; range, −1.2 to −15.4) and after RT alone (−6 mm/year; range, −1 to −256.2—Pallud et al. (23) and −4.5 mm/year; range, −17.8 to −22—Ducray et al. (22).

Nevertheless, duration of negative VDE is significantly longer after PCV followed by RT with a median of 8.2 years (range, 2.3–22.5) after PCV onset and 6.3 years (range, 0.4–22.3) after RT onset compared with 3.4 years (range, 0.8–7.7) after PCV alone (20), less than 1 year after TMZ alone (19), 1.7 years after CCNU alone (21), and 3.5 years (range, 0.4–9.8) after RT alone (22, 23). In our series, 60% of DLGG patients demonstrated an ongoing and prolonged response for more than 3.6 years without any oncological treatment compared with 2 years after PCV alone (20). First, multiple-agent chemotherapy (PCV) may improve duration of radiological response compared with single-agent chemotherapy (CCNU alone or TMZ alone). Second, the addition of PCV to RT may increase response duration by 2.8 years (20) and the addition of RT to PCV may increase response duration by 4.8 years (23). In our series, two patients benefited from this association for a long time with a persistent negative VDE 16.5 and 21.9 years after the end of PCV (without new treatment). Such prolonged responses were not reported after PCV alone or RT alone. Indeed, the median upper range observed was 7.7 years after PCV onset (20) and 9.8 years (23) or 8.1 years (22) after RT. Third, PCV associated with RT allowed a noteworthy volume response comparable with RT alone (median response rate of 65.1% (range, 9.6%–99%) in this study versus 65.9% (range, 11.7%–87%) after RT alone (23). We did not identify pattern of response as previously reported (fast and slow responders (23) or two-phase responses (22).

### Impact on Survival

In the present study, all patients were retrospectively considered at high-risk DLGGs as described in RTOG 9802: age ≥40 years with subtotal resection or biopsy irrespective of age (2). Estimated median survival since radiological diagnosis was 17.4 years (compared with 13.3 years in RTOG 9802). This subgroup of patients corresponds to the upper range of estimated median survival previously observed in our monocentric cohort of DLGG patients (median OS, 15.7 years) (7). Recent results have been published concerning the RTOG 0424 study in high-risk DLGGs (4) (risk differently defined from RTOG 9802) treated with TMZ-based chemoradiation followed by twelve cycles of TMZ. The median survival was 8.2 years. Our data corroborate that PCV chemotherapy and RT had a synergistic oncological effect with benefit on survival.

In RTOG 9802, median survival was longer in 1p19q codeleted tumors when they were treated by the combined treatment compared with RT alone: OS not reached versus 13.9 years (24). Nevertheless, molecular data were limited: 106 on 251 patients initially included were tested for molecular analysis of whom only 37 patients with 1p19q codeleted tumors (24). Concerning RTOG 0424 (4) and EORTC 22033-26033 (25) studies with TMZ-based chemotherapy, survival, and extensive molecular data are not yet available. Median survival seems to be higher in IDH-mutated/codeleted DLGGs. In this way, this subgroup is even more exposed to long-term neurotoxicity. In our series, we pointed out a better survival in patients who received a total dose of RT superior to 54 Gy whereas large previous studies demonstrated no impact of total dose of RT on survival (26). Our results may be biased due to the limited number of patients and the retrospective feature. However, KPS before the combined treatment, a well-recognized prognostic factor (27), was also significant in our study.

### Neurocognition and QOL Data

Neurocognitive assessment should be an integral part of long-term follow-up trials involving RT in DLGGs considering the risk of late cognitive decline occurring in at least 12% of patients who received RT (28). Cognitive deterioration may follow RT by several months to many years, despite limited volumes and fractions (28, 29). However, few data are available. This can be related to limited number of patients (30), concise MMSE assessment (5), or insufficient follow-up (31). Neither RTOG 9802 (2), NCCTG-RTOG-ECOG (32), nor EORTC 22844 (33) studies included formal and longitudinal neurocognitive assessments (other than MMSE) as an integral part of the trial. We will probably never get robust neurocognitive and QOL data from these prospective studies with long-term follow-up while these data seem essential. Extensive longitudinal neurocognitive data will be collected in EORTC 22033-26033 (25) and in RTOG 0424 (4) studies, evaluating TMZ-based chemotherapy. At long-term follow-up of EORTC 26951 study, 32 patients (on 368 initially included) were evaluated with a median survival of 12.3 years (9.3–14.2). A total of 69% were treated with the combined treatment. Four progression-free patients were unable to be tested neuropsychologically because of significant neurological or cognitive decline. Also, 30% suffered from severe cognitive impairment; whereas, 19% of progression-free patients needed to be institutionalized (15% in our series) and 41% were employed with or without adjusted work (30) (35% in our series). These data corroborate ours with 30% of severe cognitive decline with a median follow-up of 17.5 years leading to 25% of premature death in progression-free patients.

For surviving patients, the majority harbored significant cognitive impairment in several domains: verbal episodic memory, attention, verbal working memory, executive functions, and language abilities. Repetitive long-term neurocognitive evaluations confirmed cognitive deterioration over time, especially in verbal episodic memory, with a median delay of 24.1 years after RT completion. We probably have underestimated the presence and severity of cognitive impairment in our series, due to the lack of comprehensive and systematic neurocognitive follow-up (linked to the retrospective feature of this work). Severe central neurotoxicity has been observed after RT alone or in association with nitrosourea (34) and even after intensified PCV protocol with brain atrophy (35) that underlined the potential neurotoxicity of PCV chemotherapy alone. Indeed, CCNU and procarbazine are alkylating agents that easily cross the blood–brain barrier. Moreover, DNA adducts produced by procarbazine have been shown to potentiate nitrosourea cytotoxicity and DNA crosslinking in glioma cell lines (36). Radiation-induced cognitive impairment has been described in DLGG patients at 6 and 12 years after first diagnosis even with “safe” fraction doses (≤2 Gy). Deterioration mainly concerns attention functioning, executive functioning, and information processing speed deficits (37) as reported in our long-term survivors. However, neurotoxicity may be increased by CT given before, during, or after RT notably because of alterations in the blood–brain barrier promoted by RT. In this way, the association of CT and RT may potentiate the toxic effects of RT (28). Regarding fatigue, we reported a median score of 22.2% on EORTC QLQ-C30 and a median total score of 58% (range, 46%–65%) on MFI questionnaire for the six evaluable patients. These results seem to be more important than data described in Struik et al. (39%) (38). This symptom could play a part in QOL alteration. Indeed, in our series, global QOL was moderately impaired for the six living patients assessed (median score, 70.8%; range, 50%–83.3%). Successive QOL evaluations underlined a tendency to lower long-term scores (noticed for cognitive functioning scale).

### Place of RT, PCV, and TMZ

The optimal management of DLGGs is still debated.

The first point concerns the definition of the concept of “high risk,” which differs from one study to another (2, 4, 25, 27) leading to some confusion. The high-risk definition in RTOG 9802 takes into consideration quality of surgical removal without specifying its modalities, although functional resection with intraoperative direct cortical and subcortical electrical stimulations has clearly demonstrated a strong impact on survival (39) all the more in codeleted tumors (40). So, according to RTOG 9802, a young patient (<40 years) with subtotal removal of an IDH-mutated/codeleted tumor is considered high risk whereas his expected survival is about 14 years or more. The actual debate completely eludes the place of surgery at diagnosis and at recurrence (41) while the most robust data in terms of survival concern surgical treatment. Safe reoperation could be performed at recurrence in selected patients while preserving QOL (42).

The second point concerns the paradox of proposing an intensive treatment to a population (IDH-mutated/codeleted tumor) with an *a priori* good prognosis (long-survival expected and lower tendency to progress to more aggressive tumors), while we know the risk of late toxicity due to RT possibly increased by the association with CT. This subgroup of patients could, on the contrary, represent a population for which adjuvant RT may be delayed.

Third, neither RTOG 9802 (2) nor RTOG 0424 (4) studies integrated an arm evaluating CT alone by reserving radiotherapy for progression (if no surgery can be offered). This type of information would be essential to guide initial treatment more clearly.

Fourth, recent studies on TMZ-based chemotherapy demonstrated oncological efficacy allowing to delay RT and potential cognitive deterioration: in the UCSF study, 53% of DLGG patients did not receive RT at a median follow-up of 5.8 years (43) and in the AINO study, 48% of patients did not receive RT (58% of them were codeleted tumors), at a median follow-up of 8.2 years (44).

The last question concerns the impact of a sequential approach consisting of initial chemotherapy (TMZ or PCV) followed by reoperation and/or RT more or less chemotherapy (TMZ or PCV) at relapse compared with early RT plus PCV in terms of onco-functional balance between oncological results/survival versus cognitive and QoL preservation.

Fifth, the question persists whether PCV- or TMZ-based chemotherapy is more effective. PCV seems to have longer response duration (as described in Peyre et al. (20) and our series) and could delay RT longer than TMZ, and so the cognitive deterioration associated to RT. Nevertheless, a recent study on TMZ-based chemotherapy alone (43) found similar results to radiation arm of RTOG 9802 study in terms of inclusion criteria and survival. Another debate emerged concerning the potential risk of hypermutated status induced by early alkylating agent especially TMZ-based chemotherapy (45). The hypothesis of recurrent defects in mismatch repair (MMR) pathway driving hypermutation and secondary resistance to TMZ has been recently evoked. Interestingly, MMR-deficient cells seem to keep sensitivity to CCNU, which could argue to prefer used PCV to TMZ. Nevertheless, further studies are needed, in order to specify which patients may develop a hypermutated status induced by TMZ (46) and if hypermutated status has an impact on clinical and radiological DLGG evolution (47).

Sixth, RT could negatively impact neuroplasticity by disrupting white-matter connectivity (48), especially after maximal functional removal (up to the axonal connectivity) (49) and at the same time, promote the emergence of long-term cognitive disorders. RT, even focal, may increase risk of delayed neurotoxicity, despite improvement of techniques, according to the risk of functional connectivity injury (48, 49). Chemotherapy seems to reduce subcortical fiber infiltration rather than disrupt them and, in this way, may not interfere with neuroplasticity mechanisms (50). The challenge remains to identify the patients most at risk of long-term neurotoxicity and to optimize RT planning at individual level, in order to preserve individual neural networks. The first step could consist in redefining the classical “organs at risk,” by integrating structural and functional connectivity (51). As demonstrated in our series, neurocognitive deterioration is very heterogeneous, from severe and early cognitive impairment in 30% of patients with limited tumor volume before treatment (median tumor volume less than 35 cm^3^ before PCV and before RT) to moderate and late cognitive impact in few long-term survivors (five patients). Interestingly, these five living and active patients were never operated on, presented a median volume tumor before PCV of 118 cm^3^ (range, 68–174) and before RT of 81 cm^3^ (range, 34–138). Four patients were able to resume a professional activity and the last was not due to driving license legislation. Nevertheless, on longer follow-up, they presented a significant cognitive decline (verbal episodic memory, working memory, executive functioning, attention, language). The comprehension of neural networks and their relationship with DLGG topography is necessary to estimate the possible neurocognitive deficits that could be induced by the tumor progression and/or by the different oncological options.

## Limitations

Our study has several limitations due to its retrospective feature, small sample size, and having focused on patients treated before 2000. Thus, only printed MRI were available, requiring the use of three-diameter technique to assess tumor volume, which may lead to volume overestimation, especially with concomitant radiation-induced leukoencephalopathy (8). Molecular data were also restricted to the assessment of the canonical IDH1-R132H mutation, so it is possible that the only patient classified as IDH1wt harbored undetected IDH mutations. 1p19q codeletion was deduced from ATRX and INA expressions. MGMT promoter methylation status was not studied, although it has been previously associated with benefit to PCV therapy. However, the presence of MGMT methylation seems to be correlated with IDH mutational status (52). Finally, sixteen patients (80%) were irradiated before 2000: irradiation techniques have improved since.

## Conclusions

We reported a series of twenty DLGG patients treated early by PCV immediately followed by radiotherapy, independent of surgical status. Although this combination was associated with an oncological benefit (oncological synergy), evidenced by prolonged duration of response, substantial long-term neurotoxicity, leading to premature death in a significant number of progression-free patients (neurotoxic synergy), was highlighted. The issues are (i) to specify which patients are at risk of long-term neurotoxicity, (ii) to determine which patients are optimal candidates to postpone RT while preserving the chances of survival with the best QoL, and (iii) to better understand mechanisms of neurotoxicity and impact of treatments on neuroplasticity and connectivity. These concepts lead to involve the patient in therapeutic orientation, by informing him of the potential benefits and risks of the proposed strategy, at short, middle and long term. The optimal balance on oncological and functional features pleads in favor of a personalized dynamic therapeutic strategy.

## Data Availability Statement

The original contributions presented in the study are included in the article/supplementary material. Further inquiries can be directed to the corresponding author.

## Ethics Statement

Ethical review and approval was not required for the study on human participants in accordance with the local legislation and institutional requirements. The patients/participants provided their written informed consent to participate in this study.

## Author Contributions

Data collection: MB, TO, AB, CD, MM, LL, NF, CP, GG, and LT. Data analysis: MB, TO, and LT. Data interpretation: MB, TO, CP, GG, and LT. Report writing: MB, TO, CP, and LT. Proofreading and paper approval: MB, TO, CB, CP, CD, MM, LL, NF, AB, GG, M-HB, FR, SM, YG, AV, GV, RA, J-MM, and LT. All authors listed have made a substantial, direct, and intellectual contribution to the work and approved it for publication.

## Conflict of Interest

The authors declare that the research was conducted in the absence of any commercial or financial relationships that could be construed as a potential conflict of interest.

## Publisher’s Note

All claims expressed in this article are solely those of the authors and do not necessarily represent those of their affiliated organizations, or those of the publisher, the editors and the reviewers. Any product that may be evaluated in this article, or claim that may be made by its manufacturer, is not guaranteed or endorsed by the publisher.
